# Editorial: Liver cancer awareness month 2024: current progress and future prospects on advances in primary liver cancer investigation and treatment

**DOI:** 10.3389/fonc.2025.1737997

**Published:** 2025-11-20

**Authors:** Francisco Tustumi, Rodrigo Xavier das Neves, Marina Alessandra Pereira, Wellington Andraus, Fabrício Ferreira Coelho

**Affiliations:** 1 Universidade de São Paulo, Departamento de Gastroenterologia, São Paulo, Brazil; 2 Hospital Israelita Albert Einstein, Departamento de Ciências da Saúde, São Paulo, Brazil; 3 University of Pittsburgh, Department of Medicine, Pittsburgh, PA, United States; 4 Instituto do Câncer do Estado de São Paulo, São Paulo, Brazil

**Keywords:** liver neoplasms, hepatocellular carcinoma, cholangiocarcinoma, hepatectomy, precision medicine

Liver cancer ranks among the most lethal cancers globally, and projections indicate that its burden may rise by more than 50% over the next two decades (1). In this context, Liver Cancer Awareness Month, observed every October, provides a vital platform to discuss the disease’s global impact and to highlight recent advances in prevention, detection, and therapeutic innovation.

The most frequent primary liver cancers are hepatocellular carcinoma (HCC) and cholangiocarcinoma (2, 3). Despite improvements in surgical techniques and systemic therapies, survival rates remain low, particularly for those diagnosed at advanced stages. The need for early detection, risk stratification, and tailored therapeutic strategies has driven increased research into molecular biomarkers, immunotherapy, targeted therapies, and imaging-based innovations. These advances align with the primary goal of shifting from a one-size-fits-all approach to precision medicine, where tumor biology, host factors, and microenvironmental signatures increasingly guide treatment selection (4).

**Figure 1:**
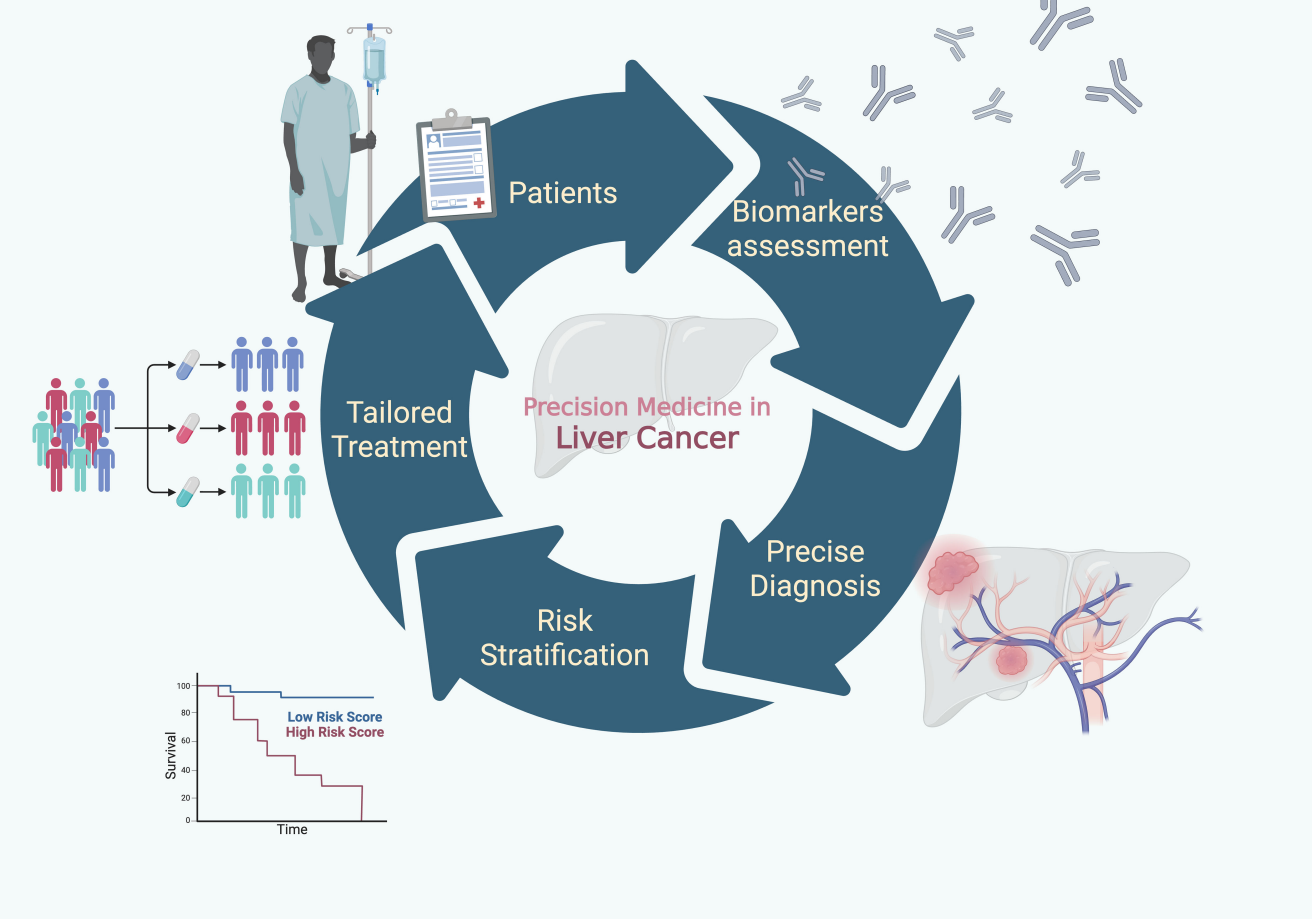
Towards precision medicine: Conceptual framework of precision medicine in primary liver cancer. The pathway from biomarker assessment to precise diagnosis, risk stratification, and tailored treatment illustrates how molecular and clinical indicators guide individualized therapeutic strategies, ultimately aiming to improve patients’ survival outcomes.

This editorial builds upon our prior initiative, which highlighted the progress in pathogenesis, diagnostic advances, and technological innovation (5). The current Research Topic on Liver Cancer Awareness Month 2024 extends this vision, featuring 25 articles that address emerging biomarkers, diagnostic strategies, and novel treatment modalities poised to individualize patient care. Together, these contributions reflect where the field is heading: towards biologically informed, patient-centered, and precision-driven approaches to combat liver cancer (**Figure 1**). The progressive shift toward precision medicine in liver cancer has been largely fueled by the discovery, validation, and clinical application of biomarkers—molecular or imaging signatures that refine diagnosis, risk stratification, and therapeutic tailoring. Traditional modalities such as imaging and histopathology remain indispensable, but they are increasingly complemented by biomarker-based approaches that allow earlier detection and more nuanced prognostic assessment, both in early and advanced disease.

Biomarkers are increasingly recognized as fundamental tools for improving the diagnosis of primary liver cancer, especially in the early stages when clinical and imaging findings alone may be insufficient. Although modern imaging modalities already provide moderate sensitivity, their specificity remains suboptimal in certain scenarios, which is critical when deciding on aggressive interventions such as hepatectomy or liver transplantation. Dong et al. reported that contrast-enhanced MRI achieved the highest diagnostic performance among imaging techniques for early HCC, yet with only moderate accuracy (sensitivity 66%, specificity 55.5%). This limitation highlights the importance of molecular characterization in refining diagnoses, ensuring appropriate treatment selection, and improving prognostic accuracy.

Several contributions within this Research Topic underscore the role of biomarkers as complements to conventional imaging. Zhao et al. described a rare case of primary hepatic carcinosarcoma where immunohistochemistry was essential to define its biphasic nature, exemplifying how pathology-guided molecular tools can overcome the limitations of radiology. Li et al. applied fluorescence lifetime imaging microscopy (FLIM) to standard H&E slides, generating quantitative metabolic signatures that distinguished tumor from peritumoral tissue and correlated with liver-functionindices.

However, biomarkers derived exclusively from surgical specimens have limited clinical utility, as they cannot support preoperative decision-making or guide treatment strategies in advance. Investigating serum markers, Feng et al. demonstrated that ESPL1 levels hold promise for the early detection of hepatitis B virus-related HCC, particularly in patients who test negative for AFP and PIVKA-II. Jiang et al. systematically evaluated liquid biopsy approaches, showing that circRNAs and mRNAs—such as hsa_circ_000224 and KIAA0101 mRNA—outperform traditional biomarkers in distinguishing HCC from both healthy controls and patients with chronic liver disease. Together, these studies reinforce the potential of biomarker-based strategies to expand diagnostic precision, promote earlier intervention, and ultimately improve patient outcomes.

Beyond their diagnostic applications, biomarkers are increasingly central to risk stratification and prognostic assessment in liver cancer. Simple serum-based indices, such as the ALBI score, are routinely used in clinical practice to predict outcomes in HCC (6, 7). They remain attractive for clinical practice due to their accessibility and cost-effectiveness. Fang et al. evaluated the HALP score (hemoglobin × albumin × lymphocytes/platelets) in HCC patients undergoing TACE and ablation. Patients with higher HALP values exhibited modestly longer recurrence-free survival, but a nomogram integrating cirrhosis, tumor number, and γ-glutamyl transpeptidase provided more accurate recurrence prediction. Similarly, Wei et al. explored dynamic perioperative biomarkers, demonstrating that changes in AST and ALT levels, combined with tumor and viral factors, could be incorporated into predictive models to identify patients at elevated risk of early recurrence after hepatectomy. Huang et al. evaluated metabolic syndrome indicators, based on clinical and serum laboratory test results, such as plasma glucose, triglycerides, and cholesterol, and found that metabolic syndrome significantly worsens survival in elderly patients with HCC.

More complex molecular indicators have also been translated into clinical tools. Xia et al. developed a nomogram based on complement C3 levels to estimate survival in early-stage HCC patients with microvascular invasion following resection. By stratifying patients into distinct prognostic categories, their model highlights how biomarker-informed algorithms can support postoperative surveillance. Ning et al. conducted a bibliometric analysis of ferroptosis research in HCC, emphasizing its rising prominence as a molecular process with prognostic and therapeutic potential. Ferroptosis is not yet measurable through routine clinical biomarkers, but current research highlights its potential to inform future prognostic models and guide the development of targeted therapies.

Imaging-derived biomarkers are also emerging as valuable prognostic tools. Li et al. showed that histogram features from diffusion-weighted imaging, particularly when combined with AFP levels, reliably predict Ki-67 expression—a well-established marker of tumor proliferation and aggressiveness (8). This integration of radiomics and molecular biomarkers exemplifies the convergence of radiology and pathology into precision medicine.

Tao et al. developed a nomogram that integrates quantitative MRI signal attenuation indices with peripheral CD4+ T-cell counts to predict response to combined systemic therapy. Lian et al. demonstrated that perfusion parameters from contrast-enhanced ultrasound could accurately predict histological differentiation of HCC, with strong diagnostic performance across both training and testing sets. Zhang et al. applied habitat imaging analysis combined with machine learning models to preoperatively predict early recurrence after hepatectomy, showing that radiomics-derived tumor subregions from CT images can capture biological heterogeneity with high predictive accuracy. Yin et al. developed and compared 2- and 3-D radiomics models from multiphase CT to predict microvascular invasion preoperatively, demonstrating that radiomics-based models consistently outperformed those relying on clinical features alone.

Integration of imaging and biochemical biomarkers also refines local therapies. Zou et al. conducted a meta-analysis showing that the combination of imaging parameters with serum biomarkers in CEUS-guided microwave ablation significantly improved complete ablation rates and reduced local recurrence.

Better treatment selection begins with better biomarkers. Ni et al. identified PDZD11 as a prognostic and diagnostic adjunct that, when combined with AFP, achieved excellent discriminatory performance. Beyond incremental accuracy, such markers provide real clinical value by addressing a fundamental question in liver cancer management: who should receive intensified systemic therapy immediately, and who may safely defer or follow a less aggressive strategy? This level of precision is particularly relevant in an era where systemic options must be tailored to maximize benefit while limiting toxicity.

In advanced and initially unresectable HCC, systemic therapy remains the backbone of treatment. Tyrosine kinase inhibitors (TKIs), such as sorafenib, target multiple signaling pathways involved in tumor growth and angiogenesis and were the first agents to demonstrate an overall survival (OS) benefit in phase III trials (median OS 10.7 vs 7.9 months compared with placebo; p<0.001) (9). More recently, immune checkpoint inhibitors (ICIs), including PD-1 and PD-L1 inhibitors, have shown efficacy by unleashing antitumor immune responses, and are now incorporated into first-line recommendations. Ding et al. reviewed how molecular knowledge from checkpoint pathways (PD-1/PD-L1, CTLA-4) to adoptive cellular therapies (CAR-T, CAR-NK, TCR-T) and macrophage-directed interventions is guiding new therapeutic options.

A range of systemic agents has emerged, including TKIs such as regorafenib and lenvatinib, as well as ICIs like nivolumab and pembrolizumab, each acting via distinct mechanisms, and combination strategies are now central in managing advanced and unresectable HCC (10, 11). Concurrently, advances in locoregional therapies, such as TACE and radioembolization, expanded treatment possibilities and created new opportunities for integration with systemic therapy. Many patients once deemed incurable may now become candidates for curative surgery after preoperative strategies designed to increase resectability (12). Bu et al. demonstrated that combining ICIs with locoregional therapy and TKI improves survival in unresectable HCC, supporting a biology-matched strategy. Xiong et al. also evaluated ICIs in combination with TKI. The authors found that TACE combined with TKI and PD-1 inhibitors has lower efficacy in HCC patients with prior TIPS. While Xiong et al. evaluated only the conventional TACE, results from Chernyshenko et al. suggest that Drug-eluting beads-TACE may achieve superior survival rates than conventional TACE.

Luo et al. evaluated HCC patients with portal vein thrombosis treated with checkpoint inhibitors in combination with TKI and compared those receiving portal vein stent implantation with those receiving external beam radiotherapy (EBRT). EBRT nearly doubled OS and showed particular benefit in VP2-type portal vein thrombosis. These findings collectively emphasize that precision medicine in HCC is not confined to systemic therapies or immunotherapies. It extends equally to other treatment options, including locoregional strategies and surgical strategies. For example, Wang et al. advocate that the choice between laparoscopic radiofrequency ablation (LRFA) or the percutaneous (PRFA) approach should be tailored to individual patient and tumor characteristics.

However, evidence suggests that even in HCC with portal vein thrombosis, surgical resection or liver transplantation, when technically feasible, can achieve superior survival compared with local ablation or systemic therapy alone (13). Yang et al. compared salvage liver transplantation (SLT) and repeat hepatectomy (RH) in more than 400 patients with recurrent HCC after resection. SLT was associated with significantly higher OS and recurrence-free survival. By contrast, in those with multiple risk factors, outcomes between SLT and RH converged. Consequently, the authors advocate for a risk stratification based on clinical and tumor characteristics for proper patient selection.

Taken together, these studies show that response evaluation in HCC is increasingly multidimensional: radiological signatures, immune profiling, procedural refinements, and recurrence-risk stratification all converge to inform treatment continuation, escalation, or change. Precision medicine, therefore, lies not only in choosing the right therapy but also in continuously measuring and refining its effectiveness in the individual patient.
